# Targeted Analysis of 46 Bile Acids to Study the Effect of Acetaminophen in Rat by LC-MS/MS

**DOI:** 10.3390/metabo10010026

**Published:** 2020-01-07

**Authors:** Vivaldy Prinville, Leanne Ohlund, Lekha Sleno

**Affiliations:** Department of Chemistry, Université du Québec à Montréal (UQAM), P.O. Box 8888 Downtown Station, Montreal, QC H3C 3P8, Canada; vivaldyp@hotmail.com (V.P.); ohlund@gmail.com (L.O.)

**Keywords:** bile acids, metabolomics, rat plasma, tandem mass spectrometry, liquid chromatography, acetaminophen, hepatotoxicity

## Abstract

Bile acids represent a large class of steroid acids synthesized in the liver and further metabolized by many bacterial and mammalian enzymes. Variations in bile acid levels can be used as a measure of liver function. There still exists, however, a need to study the variation of individual circulating bile acids in the context of hepatotoxity or liver disease. Acetaminophen (APAP), a drug commonly taken to relieve pain and decrease fever, is known to cause acute liver failure at high doses. We have developed a targeted liquid chromatography-tandem mass spectrometry method to monitor the effects of different doses of APAP on the bile acid plasma profile in a rat model. The analysis method was optimized to ensure chromatographic resolution of isomeric species using a mixture of 46 standard bile acids, and 14 isotopically-labeled internal standard (IS) compounds detected in multiple reaction monitoring (MRM) mode on a triple quadrupole mass spectrometer. Four doses of acetaminophen were studied, the highest of which shows signs of hepatotoxicity in rats. This targeted method revealed that high dose APAP has an important effect on bile acid profiles. Changes were seen in several unconjugated bile acids as well as glycine conjugates; however, no obvious changes were apparent for taurine-conjugated species.

## 1. Introduction

Bile acids play many roles crucial for metabolism and liver health. They are formed from cholesterol through a series of enzymatic reactions and they represent the primary pathway for cholesterol catabolism [1]. In addition, bile acids emulsify fat from our diet and help absorb lipids and cholesterol [2]. Primary bile acids, such as cholic acid (CA) and chenodeoxycholic acid (CDCA) in humans and α-muricholic acid (α-MCA) and β-muricholic acid (β-MCA) in rodents, are synthesized in the liver. Before being secreted by the liver, bile acids can be conjugated to taurine or glycine amino acids. In the intestines, bile acids are unconjugated and converted into secondary bile acids, such as deoxycholic acid (DCA) and lithocholic acid. Most bile acids are reabsorbed in the liver, conjugated again, and excreted in the bile to complete the enterohepatic circulation [2,3].

An increased plasma concentration of bile acids is a sign of liver disease [4]. High concentrations are toxic, though the potential for toxicity depends on the bile acid profile. For example, it has been reported that chenodeoxycholic acid and lithocholic acid, as well as their conjugates, can damage hepatic cells and induce mitochondrial malfunction, oxidative stress, and apoptosis [5,6,7,8]. Bile acids can also damage cells within the colon [9,10]. The different physiological functions of bile acids and their implication in pathological processes highlight the importance of understanding circulating bile acid profiles in drug-induced hepatotoxicity.

Acetaminophen (APAP) is a drug commonly used to relieve pain and decrease fever. When taken in therapeutics doses, APAP is considered a very safe drug. With excessive doses, APAP can become highly toxic [11]. In North America, it is the main cause of acute liver failure, and often requires liver transplantation if too severe or not treated rapidly enough [12]. In extreme cases, APAP toxicity can cause death within 48 h. Previous studies have shown APAP interferes with bile acid synthesis [13,14,15].

Different LC-MS based methods to measure bile acids exist [16,17], but a gap still remains with regards to the wide range of bile acid derivatives that exist and their changing profiles with APAP dose. By studying the effect on individual bile acids, specific reactions related to bile acid metabolism can be assessed as being relevant to follow altered hepatic metabolism. The goal of this study was to develop an optimized and semi-quantitative method to evaluate the effects of APAP on numerous bile acids, including free and conjugated forms. Liquid chromatography coupled to tandem mass spectrometry is a powerful technique that offers many advantages for selective detection of individual bile acids, which are uniquely challenging due to the presence of many isomers. Bile acids can be difficult to analyze due to the similarities between the structures. In this study, we developed a rapid method to monitor 46 bile acids by LC-MS/MS on a triple quadrupole platform in multiple reaction monitoring (MRM) mode.

## 2. Results and Discussion

**Figure 1:**
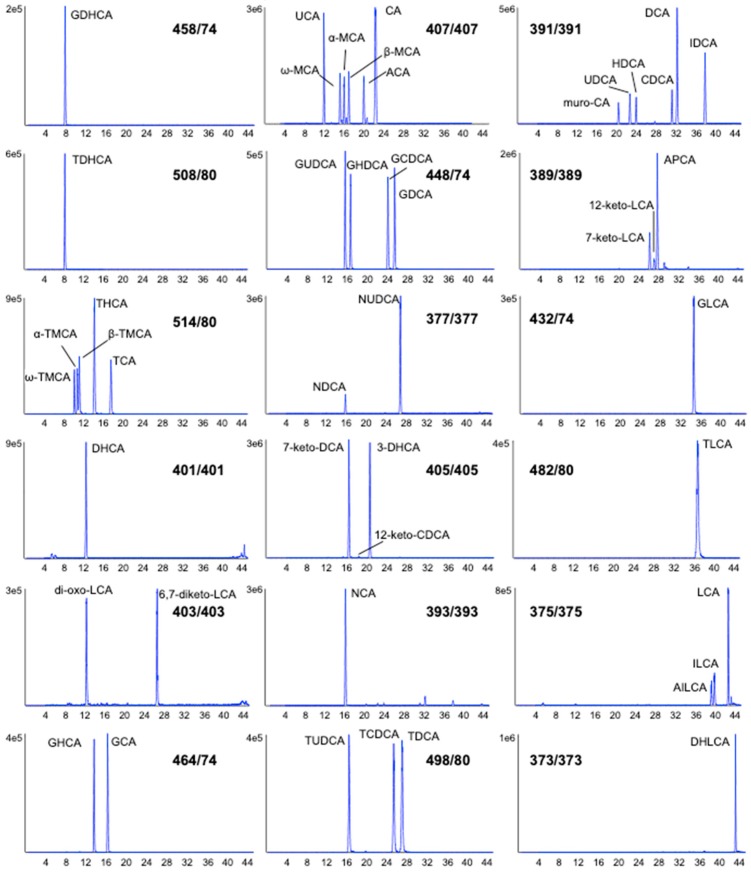
Representative LC-MRM chromatograms in negative mode of a standard mixture containing 46 bile acids (using the most sensitive transition for each bile acid, as shown). Acronyms for each bile acid species are listed in the Abbreviations (and Methods) section.

**Figure 2:**
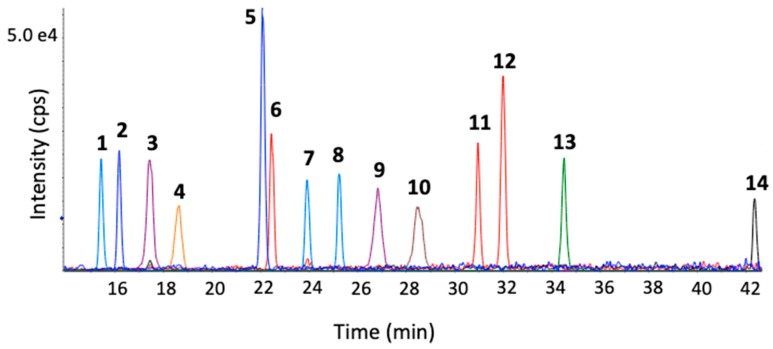
Representative LC-MRM chromatograms from a 20 μL injection of IS mix containing 0.013-0.1 μM final concentration of each IS compound. (1) d4-GUDCA (2) d4-GCA (3) d4-TUDCA (4) d4-TCA (5) d4-CA (6) d4-UDCA (7) d4-GCDCA (8) d4-GDCA (9) d4-TCDCA (10) d6-TDCA (11) d4-CDCA (12) d4-DCA (13) d4-GLCA (14) d4-LCA.

A targeted liquid chromatography-multiple reaction monitoring (LC-MRM) method was developed to monitor 46 bile acids in rat plasma following a simple sample preparation to evaluate the effect of increasing APAP dose. Bile acids were extracted by protein precipitation using methanol, following the addition of an isotopically labeled internal standard mix. A reverse-phase solid-core C18 column was employed to separate the 46 bile acids with excellent resolution and peak shape using acidified water and acetonitrile as mobile phase, within a 45 min gradient. As shown in Figure 1, all 46 bile acids in the standard mix were well resolved, including many bile acid isomers (e.g., UDCA, CDCA, and DCA). For example, LC-MRM chromatograms for α-TMCA, β-TMCA, and TCA in rat plasma show good resolution obtained and highlight the usefulness of this method to monitor these isomers. The list of bile acids assessed in this study was based on the availability of a standard mix as well as multiple isotopically-labeled bile acids for relative quantitation, through a generous gift from MRM Proteomics Inc. The separation of these internal standard (IS) compounds is shown in Figure 2.

LC-MRM analyses in negative ion mode yielded better results than in positive ion mode in terms of sensitivity (data not shown), though both were optimized. In positive mode, precursor ions were often associated to in-source water losses and had limited sensitivity as compared to negative mode. In negative mode, unconjugated bile acids were monitored with two transitions, the highest signal coming from monitoring the pseudo-MRM transition of precursor ion to precursor ion, since their fragmentation resulted in a complex mix of fragments, thus limiting sensitivity for more specific fragment ions [18]. For conjugated bile acids, fragment ions resulting from the taurine and glycine moieties were employed as product ions. For each bile acid, however, secondary transitions were monitored for confirmatory purposes. In rat plasma samples, 39 of the 46 bile acids were measurable, with peaks having signal-to-noise of at least 10 and retention time matching that of the standard mix. No peak was observed for GDHCA, TDHCA, IDCA, DHCA, TLCA, AILCA and ILCA in rat plasma samples. DHCA is a synthetic product of the oxidation of CA and is mainly converted into 3-α-hydroxylated-oxo bile acids [19]. It is therefore normal that the conjugated bile acids of DHCA (GDHCA and TDHCA) are not present in rat plasma either. Iso-bile acids (IDCA, AILCA and ILCA) are excreted in the feces of animals [20]. Of the 39 bile acids remaining, several had very small peaks that did not yield any statistically-significant changes between APAP doses, including GHCA, GLCA, GUDCA, NCA, NUDCA, DHLCA, LCA, di-oxo-LCA and 6,7 diketo-LCA.

**Figure 3:**
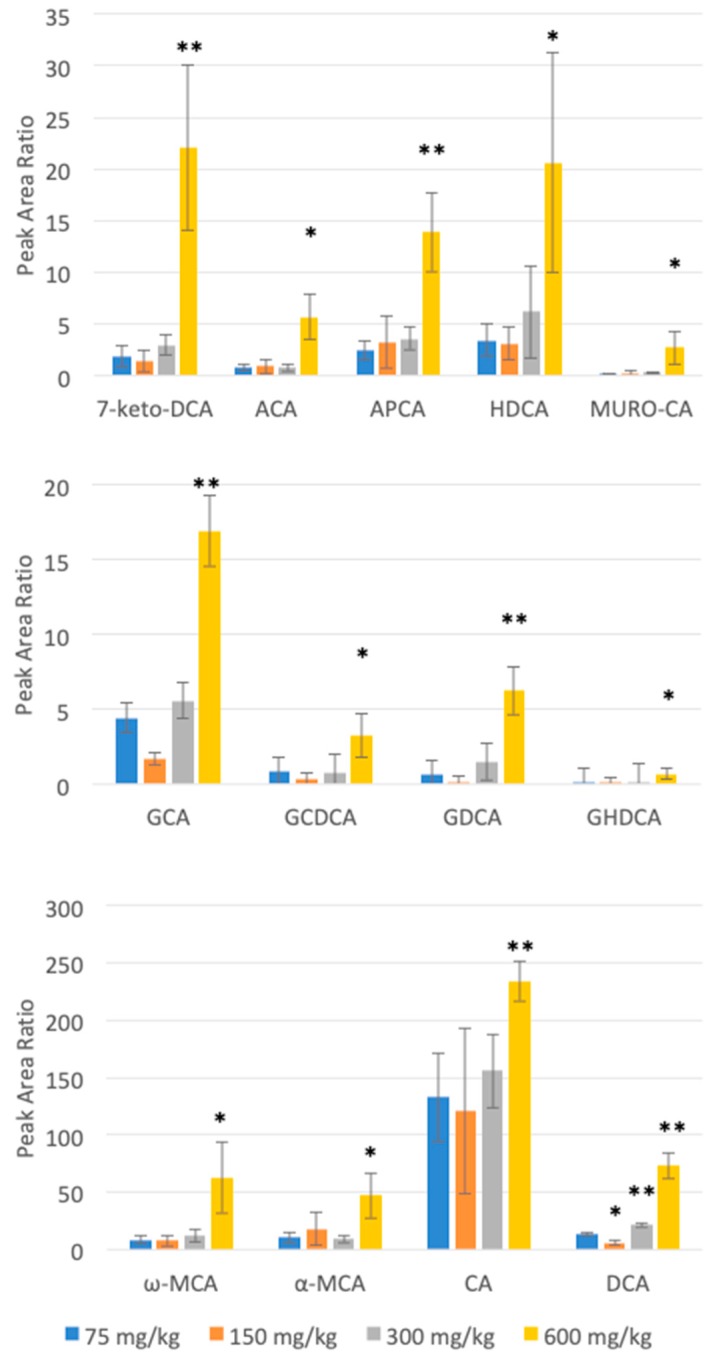
Peak area ratios for bile acids having significant changes between 75 mg/kg and 600 mg/kg APAP dosing, as measured in rat plasma after 24 h. * *p*-value < 0.05, ** *p*-value < 0.01.

The highest APAP dose administered in this study significantly influenced the peaks corresponding to several bile acids (Figure 3). Table S1 shows the *p*-values and fold changes seen for each of these changing bile acids at each of the dosing levels compared to the lowest dose. This table also shows the integration data considering both MRM transitions monitored for each of these bile acids, and confirms that for all except two which were too small to properly integrate, these secondary transitions correlated well with the first (more sensitive) transition. Each MRM peak was also investigated for saturation effects. Although no linear ranges were determined directly, based on the peak heights of these bile acids, it was confirmed that we would be able to detect changes in terms of fold change (up or down). It is, however, very important to state here that fold changes of peak area ratios do not directly translate into concentration fold changes. These results are reported to determine which bile acids of the 46 from the standard mix were well observed in rat plasma samples and which were altered significantly with increased APAP dose. Thirteen bile acids of the 30 having significant signal-to-noise in our samples were shown to have statistically-relevant changes between the lowest and highest dose given in this study, with a *p*-value of lower than 0.05, six of which had *p*-values lower than 0.01. Increasing the APAP dose affected the concentration of some bile acids more than others. The bile acids with the most significant changes (with *p* < 0.01) were GCA, GDCA, 7-keto-DCA, APCA, CA and DCA. The graphs in Figure 3 show the peak area ratios at all four doses of APAP. The taurine conjugates monitored did not show any statistically relevant changes with APAP dose. An important effect was seen, however, for several conjugated glycine conjugates. All four glycine conjugates having adequate peak size (GCA, GCDCA, GDCA and GHDCA) were found to significantly increase between 75 and 600 mg/kg APAP. The three with less obvious quantitative changes were notably much smaller peaks in the rat plasma extracts. For example, the peak area ratio for GDCA was 10.1 times higher (with a *p*-value of 0.0024) with 600 mg/kg compared with 75 mg/kg APAP, while the corresponding taurine conjugate, TDCA, did not show any effect at the highest dose. Since the conjugation of bile acids is an important pathway for their secretion by the liver, our results indicate that APAP could influence the transfer pathway of bile acids from the liver to the bloodstream.

We found that for the two primary bile acids, CDCA and CA, only CA was found to have a statistically significant increase with APAP dose levels (fold change of 1.8 and *p*-value of 0.005 at highest dose). Peak area ratios for α-MCA, and ω-MCA had increased by 4.6-, and 7.4-fold (*p*-value of 0.0268, and 0.0322), respectively. Given that CDCA is transformed by 6β-hydroxylase in rat liver into α-MCA, β-MCA, and ω-MCA, it is likely that CDCA is mostly converted into different MCA isomers [21]. The peak area ratio of DCA increased 5.6-fold, (with a *p*-value of 0.0003). Interestingly, DCA has been reported to induce both early apoptosis and necrosis, thus affecting cell development [22]. The fold changes between different individual bile acids cannot be directly compared, of course, since the relative response and sensitivity of each compound by LC-MS/MS is unique. We are not assuming that a larger fold change from this data set gives a stronger change in actual concentration. This would need a follow-up study for absolute quantitation of individual bile acids, with calibration curves for each. This is quite difficult, however, considering we are not able to construct traditional calibration curves for endogenous metabolites in complex biological matrices, such as plasma, as is done for therapeutic drug monitoring.

**Figure 4:**
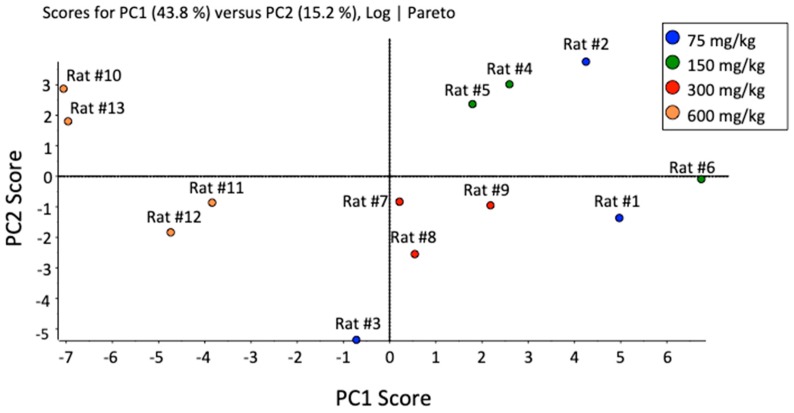
Unsupervised principal component analysis (PCA) of 39 bile acids detected in rat plasma samples (comparing four APAP dosing groups).

The LC-MRM data was imported into metabolomics software (MarkerView^TM^) to perform statistical analyses (Student’s t-test, as shown previously) and also to visualize data presented within a principal component analysis (PCA). Figure 4 shows the PCA plot of the first two principal components (PC1 vs. PC2), with Pareto scaling to alleviate bias to highest peaks. This plot shows clearly that the highest dose of 600 mg/kg clusters separately to the three lower doses (75, 150, and 300 mg/kg), as was evident from the t-testing results of the individual bile acids. The PCA plot, which used all features from the LC-MRM data, following supervised peak integration, serves to show that the high dose had a marked effect compared to the three lower doses, instead of seeing a gradual shift between the four doses.

A higher throughput method could be devised to assess the specific bile acids perturbed by APAP in a follow-up study, for a more rapid assessment of changes in a clinical setting, for instance. It is important to note, however, that there exists many isomers of bile acids in biological samples and that even if we are interested in targeting a finite list of specific ones for a follow-up assay, we would still need to ensure proper separation of all these isomers. The study presented here focused specifically on evaluation the 46 bile acids available from a known standard mix. This method is not presented for the purpose of being a clinical assay, since it would likely not be high throughput enough considering the chromatographic separation needed to access all these different isomers. It also does not serve to accurately quantify each bile acid (in terms of concentration), rather it looks at relative amounts of bile acids (e.g., their profiles) in a biological matrix (rat plasma) to look for specific effects of APAP dose on individual bile acids. Therefore, this work should not be considered as a new validated method, as per US FDA guidelines. It would be interesting in a future study to validate a method for the bile acids specifically perturbed by high dose APAP. This is quite challenging in the case of endogenous metabolites since it would necessitate stable isotope standards for each metabolite to be quantified, as well as a suitable “blank” biological matrix to be used for preparing calibration curves for each analyte. Additionally, a non-targeted metabolomics approach using high-resolution tandem mass spectrometry would be able to access many more bile acid isomers, as well as sulfate and glucuronide metabolites, without the need for optimizing MS/MS parameters for MRM detection.

## 3. Materials and Methods

### 3.1. Materials

HPLC-grade acetonitrile (ACN) and methanol (MeOH), as well as LC-MS-grade formic acid were purchased from Sigma-Aldrich (Oakville, ON, Canada). Purified water was prepared in-house. MetaboloMetrics™ bile acids analysis kits were obtained from MRM Proteomics Inc. (Montreal, QC, Canada). Kits contained a mix of 46 bile acids and an IS mix of 14 deuterated isotope-labeled internal standards. Sprague-Dawley rats were dosed (IP) with 75, 150, 300, and 600 mg/kg APAP, in triplicate, and plasma was collected after 24 h at INRS Centre de Biologie Experimentale (Laval, QC, Canada), within standard ethical practices. The protocol was approved by the Ethics Committee of the INRS Centre de Biologie Experimentale under the ethical practices of the Canadian Council on Animal Care (project UQLK.14.02). These samples were collected in February 2014 and stored at −80 °C until proceeding with sample preparation.

- The standard mix containing 46 bile acids (each at 2.5 nmol), except for deoxycholic acid (5 nmol) and taurohyocholic acid (6.5 nmol) was provided as a dried sample (Tube A). The bile acids in the standard mix were as follows: glycodehydrocholic acid (GDHCA), taurodehydrocholic acid (TDHCA), tauro-ω-muricholic acid (ω-TMCA), tauro-α-muricholic acid (α-TMCA), tauro-β-muricholic acid (β-TMCA), taurohyocholic acid (THCA), taurocholic acid (TCA), dehydrocholic acid (DHCA), dioxolithocholic acid (di-oxo-LCA), 6,7-diketolithocholic acid (6,7-diketo-LCA), glycohyocholic acid (GHCA), glycocholic acid (GCA), ursocholic acid (UCA), ω-muricholic acid (ω-MCA), α-muricholic acid (α-MCA), β-muricholic acid (β-MCA), allocholic acid (ACA), cholic acid (CA), glycoursodeoxycholic acid (GUDCA), glycohyodeoxycholic acid (GHDCA), glycochenodeoxycholic acid (GCDCA), glycodeoxycholic acid (GDCA), nordeoxycholic acid (NDCA), norursodeoxycholic acid (NUDCA), 7-ketodeoxycholic acid (7-keto-DCA), 12-ketodeoxycholic acid (12-keto-DCA), 3-dehydrocholic acid (3-DHCA), norcholic acid (NCA), tauroursodeoxycholic acid (TUDCA), taurochenodeoxycholic acid (TCDCA), taurodeoxycholic acid (TDCA), murocholic acid (muro-CA), ursodeoxycholic acid (UDCA), hyodeoxycholic acid (HDCA), chenodeoxycholic acid (CDCA), deoxycholic acid (DCA), isodeoxycholic acid (IDCA), 7-ketolithocholic acid (7-keto-LCA), 12-ketolithocholic acid (12-keto-LCA), apocholic acid (APCA), glycolithocholic acid (GLCA), taurolithocholic acid (TLCA), alloisolithocholic acid (AILCA), isolithocholic acid (ILCA), lithocholic acid (LCA) and dehydrolithocholic acid (DHLCA).
- Isotopically labeled bile acids were provided as an IS mix for normalization purposes. The labeled bile acids were present at between 0.1–0.75 nmol, as a dried sample (Tube B). The labeled bile acids in the IS mix were as follows: glycoursodeoxycholic acid-d_4_ (d_4_-GUDCA), glycocholic acid-d_4_ (d_4_-GCA), tauroursodeoxycholic acid-d_4_ (d_4_-TUDCA), taurocholic acid-d_4_ (d_4_-TCA), cholic acid-d_4_ (d_4_-CA), ursodeoxycholic acid-d_4_ (d_4_-UDCA), glycochenodeoxycholic acid-d_4_ (d_4_-GCDCA), glycodeoxycholic acid-d_4_ (d_4_-5GDCA), taurochenodeoxycholic acid-d_4_ (d_4_-TCDCA), taurodeoxycholic acid-d_6_ (d_6_-TDCA), chenodeoxycholic acid-d_4_ (d_4_-CDCA), deoxycholic acid-d_4_ (d_4_-DCA), glycolithocholic acid-d_4_ (d_4_-GLCA) and lithocholic acid-d_4_ (d_4_-LCA).

### 3.2. Sample Preparation

For the standard mix preparation, 250 µL ACN was added to Tube A, followed by a 1:10 dilution with 40% ACN. This standard mix was injected (2 μL) with the LC-MRM method described (shown in Figure 1) for retention time matching of bile acids in samples. For the internal standard (IS) mix, 7.5 mL of 40% ACN was added to Tube B. Plasma samples (50 μL) were mixed with 50 μL of the reconstituted (Tube B) internal standard solution. The extraction of bile acids from rat plasma was then performed by adding 300 μL MeOH to precipitate proteins. Samples were vortexed and sonicated for 15 min, then centrifuged at 14,000 rpm for 8 min. Supernatants (300 μL) were transferred to new microtubes, dried under nitrogen and reconstituted with 150 μL 50% MeOH prior to analysis. Extracts were stored at −20 °C until LC-MS analysis.

### 3.3. LC-MS/MS

Extracted plasma and standards were separated on an Aeris^TM^ PEPTIDE XB-C18 column (1.7 µm, 100 mm × 2.1 mm) (Phenomenex^®^, Torrance, CA, USA) using a Nexera ultra high performance liquid chromatography (UHPLC) system (Shimadzu, Columbia, MD, USA) at 50 °C with gradient elution using water and ACN, each containing 0.1% formic acid as mobile phase A and B, respectively, at a flow rate of 0.400 mL/min and injection volume of 15 μL. The gradient started at 10% B and was held for 1 min increased linearly to 25% for 2 min, to 35% over 17 min, to 50% over 20 min, to 60% over 2 min and 90% for 1 min, followed by a 10 min column re-equilibration time at starting conditions. MS data was collected using a QTRAP 5500 system (Sciex, Concord, ON, Canada). Electrospray ionization (ESI) in negative ion mode and multiple reaction monitoring (MRM) was used. The MRM parameters (first and second transitions with collision energies (CE)) for all 46 bile acids are listed in Table 1). Each transition was monitored throughout the chromatogram with a dwell time of 7 ms. Because of the fragmentation behavior of the deprotonated unconjugated bile acids, the first transitions chosen for the unconjugated forms were simply precursor to precursor ions, as has been done in many previous reports on bile acid analysis [17,23,24,25]. This was necessary for ensuring the best sensitivity of detection for these unconjugated forms. For glycine conjugated bile acids, there was a specific and sensitive common fragment ion at *m*/*z* 74, corresponding to the deprotonated glycine moiety being lost. Similarly, taurine conjugates yielded a common fragment at *m*/*z* 80, corresponding to the HSO_3_^−^ ion from the taurine group. The secondary transitions were used for confirmation. For those bile acids found to be altered significantly upon APAP dose, the secondary transition was also integrated and compared.

### 3.4. Statistical Analysis

A mixture of 14 deuterated bile acids was added to the plasma samples prior to metabolite extraction, for normalization of data as peak area ratios (analyte/IS) (see Table 2). For those bile acids without corresponding deuterated analogs, the closest eluting deuterated analog was used as IS, as noted in Table 1. Standards were used to confirm the identity of each bile acid, based on retention and MRM signal. Peak integration was performed using Multiquant™ 2.1 (Sciex). Statistical analyses were done using MarkerView™ 1.2.1 (Sciex). This software was used to perform Student’s t-tests, yielding *p*-values and fold changes between different dosing groups, for each bile acid detected in rat plasma samples. Within Markerview software, principal component analysis was performed on the integrated LC-MRM data, without weighting and using Pareto scaling (unsupervised).

## 4. Conclusions

In this study, we have developed a targeted metabolomics method to gain a better understanding of the effects of APAP on circulating bile acid profiles. A simple protein precipitation procedure in rat plasma was employed rapidly prepare samples for analysis. A standard mix of 46 bile acids was successfully resolved by LC-MRM, 39 of which were detected in rat plasma samples. These analyses highlighted significant changes in bile acid profiles with increasing APAP dose in rats. In general, these results indicate that APAP can have an important effect on the metabolism of bile acids. Depending on the dose level, exposure to high or repeated APAP doses has the potential to induce serious health problems, related to bile acid metabolism and excretion. The specificity of these biomarkers to APAP-related toxicity would still need to be investigated. Certain of these bile acids can also serve as biomarkers to establish the level of hepatotoxicity; however, more work would be needed to validate specific bile acid biomarkers for clinical use.

## Figures and Tables

**Table 1:** Optimized MRM transitions and collision energies for 46 standard bile acids along with their respective retention times.

Bile Acid	RT (min)	1st Transition (CE)	2nd Transition (CE)	IS
GDHCA	7.9	458.2/74.0 (−66)	458.2/348.1 (−41)	CDCA-d_4_
TDHCA	8.2	508.2/80.0 (−123)	508.2/124.1 (−67)	CDCA-d_4_
T-ω-MCA	10.1	514.2/80.0 (−135)	514.2/107.0 (−82)	TCA-d_4_
T-α-MCA	10.7	514.2/80.0 (−135)	514.2/107.0 (−82)	TCA-d_4_
T-β-MCA	11.1	514.2/80.0 (−135)	514.2/124.0 (−65)	TCA-d_4_
UCA	11.9	407.2/407.2 (−15)	407.2/343.1 (−46)	CA-d_4_
di-oxo-LCA	12.3	403.2/403.2 (−18)	403.2/385.2 (−40)	CDCA-d_4_
DHCA	12.4	401.2/401.2 (−18)	401.2/331.1 (−36)	CDCA-d_4_
GHCA	13.6	464.2/74.0 (−82)	464.2/354.1 (−56)	GCA-d_4_
THCA	14.1	514.2/80.0 (−135)	514.2/107.0 (−82)	TCA-d_4_
ω-MCA	15.1	407.2/407.2 (−15)	407.2/371.1 (−43)	CA-d_4_
GUDCA	15.5	448.2/74.0 (−83)	448.2/386.1 (−59)	GUDCA-d_4_
NDCA	15.8	377.2/377.2 (−15)	377.2/331.1 (−46)	CDCA-d_4_
α- MCA	16.0	407.2/407.2 (−15)	407.2/371.1 (−43)	CA-d_4_
NCA	16.1	393.2/393.2 (−15)	393.2/375.1 (−45)	CDCA-d_4_
GCA	16.2	464.2/74.0 (−82)	464.2/402.1 (−46)	GCA-d_4_
7-keto-DCA	16.4	405.2/405.2 (−18)	405.2/289.1 (−51)	CDCA-d_4_
TUDCA	16.4	498.2/80.0 (−130)	498.2/107 (−82)	TUDCA-d_4_
GHDCA	16.6	448.2/74.0 (−83)	448.2/386.1 (−59)	GUDCA-d_4_
β-MCA	16.9	407.2/407.2 (−15)	407.2/371.1 (−43)	CA-d_4_
TCA	17.5	514.2/80.0 (−135)	514.2/124.0 (−65)	TCA-d_4_
12-keto-CDCA	18.4	405.2/405.2 (−18)	405.2/387.1 (−45)	CDCA-d_4_
ACA	19.9	407.2/407.2 (−15)	407.2/371.1 (−43)	CA-d_4_
muro-CA	20.3	391.2/391.2 (−15)	391.2/343.1 (−53)	UDCA-d_4_
3-DHCA	20.5	405.2/405.2 (−18)	405.2/289.1 (−51)	CDCA-d_4_
CA	22.2	407.2/407.2 (−15)	407.2/343.1 (−46)	CA-d_4_
UDCA	22.6	391.2/391.2 (−15)	391.2/373.2 (−48)	UDCA-d_4_
HDCA	23.9	391.2/391.2 (−15)	391.2/373.2 (−48)	CDCA-d_4_
GCDCA	24.0	448.2/74.0 (−83)	448.2/404.2 (−46)	GCDCA-d_4_
TCDCA	25.3	498.2/80.0 (−130)	498.2/124 (−64)	TCDCA-d_4_
GDCA	25.3	448.2/74.0 (−83)	448.2/404.2 (−46)	GDCA-d_4_
7-keto-LCA	26.1	389.2/389.2 (−18)	389.2/354.1 (−43)	LCA-d_4_
6,7-diketo-LCA	26.5	403.2/403.2 (−18)	403.2/347.1 (−39)	LCA-d_4_
NUDCA	26.7	377.2/377.2 (−15)	377.2/359.1 (−45)	CDCA-d_4_
TDCA	27.0	498.2/80.0 (−130)	498.2/124 (−64)	TDCA-d_6_
12-keto-LCA	27.1	389.2/389.2 (−18)	389.2/354.1 (−43)	LCA-d_4_
APCA	27.6	389.2/389.2 (−18)	389.2/371.1 (−43)	CDCA-d_4_
CDCA	31.1	391.2/391.2 (−15)	391.2/373.2 (−48)	CDCA-d_4_
DCA	32.1	391.2/391.2 (−15)	391.2/343.1 (−53)	DCA-d_4_
GLCA	34.6	432.2/74.0 (−66)	432.2/388.1 (−45)	GLCA-d_4_
TLCA	36.6	482.2/80.0 (−135)	482.2/107 (−80)	LCA-d_4_
IDCA	37.8	391.2/391.2 (−15)	391.2/345.1 (−45)	DCA-d_4_
AILCA	39.1	375.2/375.2 (−15)	375.2/45 (−50)	LCA-d_4_
ILCA	39.7	375.2/375.2 (−15)	375.2/45 (−50)	LCA-d_4_
LCA	42.5	375.2/375.2 (−15)	375.2/45 (−50)	LCA-d_4_
DHLCA	43.1	373.2/373.2 (−18)	373.2/45 (−50)	LCA-d_4_

**Table 2:** Optimized MRM transitions and collision energies for 14 internal standards compounds, along with their respective retention times.

	RT (min)	Q1 (*m*/*z*)	Q3 (*m*/*z*)	CE (V)
GUDCA-d_4_	15.5	452.3	74.0	−41
GCA-d_4_	16.2	468.3	74.0	−45
TUDCA-d_4_	17.4	502.3	80.0	−73
TCA-d_4_	18.7	518.3	80.0	−80
CA-d_4_	22.1	411.3	411.3	−15
UDCA-d_4_	22.5	395.3	395.3	−15
GCDCA-d_4_	23.8	452.3	74.0	−37
GDCA-d_4_	25.3	452.3	74.0	−41
TCDCA-d_4_	26.8	502.3	80.0	−80
TDCA-d_6_	28.7	504.3	80.0	−80
CDCA-d_4_	31	395.3	395.3	−15
DCA-d_4_	32	395.3	395.3	−15
GLCA-d_4_	34.5	436.3	74.0	−41
LCA-d_4_	42.4	379.3	379.3	−15

## Supplementary Materials

The following are available online at https://www.mdpi.com/2218-1989/10/1/26/s1, Table S1: Comparison of p-values and fold-changes between the four doses of APAP administered in this study (using two MRM transitions for each peak) of known bile acids showing significant differences between the lowest and highest dose APAP.

## Abbreviations

GDHCA	glycodehydrocholic acid
TDHCA	taurodehydrocholic acid
ω-TMCA	tauro-ω-muricholic acid
α-TMCA	tauro-α-muricholic acid
β-TMCA	tauro-β-muricholic acid
THCA	taurohyocholic acid
TCA	taurocholic acid
DHCA	dehydrocholic acid
di-oxo-LCA	dioxolithocholic acid
6,7-diketo-LCA	6,7-diketolithocholic acid
GHCA	glycohyocholic acid
GCA	glycocholic acid
UCA	ursocholic acid
ω-MCA	ω-muricholic acid
α-MCA	α-muricholic acid
β-MCA	β-muricholic acid
ACA	allocholic acid
CA	cholic acid
GUDCA	glycoursodeoxycholic acid
GHDCA	glycohyodeoxycholic acid
GCDCA	glycochenodeoxycholic acid
GDCA	glycodeoxycholic acid
NDCA	nordeoxycholic acid
NUDCA	norursodeoxycholic acid
7-keto-DCA	7-ketodeoxycholic acid
12-keto-DCA	12-ketodeoxycholic acid
3-DHCA	3-dehydrocholic acid
NCA	norcholic acid
TUDCA	tauroursodeoxycholic acid
TCDCA	taurochenodeoxycholic acid
TDCA	taurodeoxycholic acid
muro-CA	murocholic acid
UDCA	ursodeoxycholic acid
HDCA	hyodeoxycholic acid
CDCA	chenodeoxycholic acid
DCA	deoxycholic acid
IDCA	isodeoxycholic acid
7-keto-LCA	7-ketolithocholic acid
12-keto-LCA	12-ketolithocholic acid
APCA	apocholic acid
GLCA	glycolithocholic acid
TLCA	taurolithocholic acid
AILCA	alloisolithocholic acid
ILCA	isolithocholic acid
LCA	lithocholic acid
DHLCA	dehydrolithocholic acid
