# Supplementary material for: Targeted Analysis of 46 Bile Acids to Study the Effect of Acetaminophen in Rat by LC-MS/MS
Source: Metabolites. 2020 Jan 7;10(1):26. doi: 10.3390/metabo10010026 (PMC7022647; doi:10.3390/metabo10010026)
Supplement: Supplementary file 1 [file metabolites-10-00026-s001.pdf]

metabolites

MDPI

Article

Targeted Analysis of 46 Bile Acids to Study the Effect of Acetaminophen in Rat by LC-MS/MS

Vivaldy Prinville, Leanne Ohlund and Lekha Sleno *

Department of Chemistry, Université du Québec à Montréal (UQAM), PO Box 8888 Downtown Station, Montreal, QC H3C 3P8, Canada; vivaldyp@hotmail.com (V.P.); ohlund@gmail.com (L.O.)
* Correspondence: sleno.lekha@uqam.ca

Supplemental material

Table S1. Comparison of *p*-values and fold-changes between the four doses of APAP administered in this study (using two MRM transitions for each peak) of known bile acids showing significant differences between the lowest and highest dose APAP.

Bile Acid	transition	*p*-value*			Fold Change		
		150/75	300/75	600/75	150/75	300/75	600/75
7-keto-DCA	405/405	0.6675	0.2508	**0.0081**	0.8	1.6	11.9
	405/289	0.4568	0.4210	**0.0020**	0.7	1.3	7.9
ACA	407/407	0.8053	0.9335	**0.0136**	1.2	1.0	7.1
	407/371[a]						
APCA	389/389	0.6377	0.2350	**0.0043**	1.3	1.5	5.7
	389/371	0.7860	0.2221	**0.0071**	1.2	1.6	5.7
HDCA	391/391	0.8080	0.3706	**0.0421**	0.9	1.8	6.0
	391/373	0.3062	0.5137	**0.0302**	0.4	1.8	8.5
MURO-CA	391/391	0.7286	0.0927	**0.0455**	1.2	1.7	13.9
	391/343[a]						
GCA	464/74	0.2477	0.6603	**0.0022**	0.4	1.3	3.8
	464/402	0.2498	0.6622	**0.0059**	0.4	1.3	3.4
GCDCA	448/74	0.3071	0.8691	**0.0499**	0.4	0.9	3.9
	448/404	0.3633	0.9288	**0.0360**	0.2	1.1	6.1
GDCA	448/74	0.2312	0.0751	**0.0024**	0.2	2.4	10.1
	448/404	0.2169	0.0552	**0.0025**	0.2	2.6	10.8
GHDCA	448/74	0.3884	0.5019	**0.0398**	0.4	1.7	7.2
	448/386	0.8624	0.3002	**0.0412**	0.8	2.8	11.0
ω-MCA	407/407	0.7768	0.4250	**0.0322**	0.9	1.4	7.4
	407/371	0.7277	0.4623	**0.0240**	0.9	1.2	6.7
α-MCA	407/407	0.4266	0.6960	**0.0268**	1.8	0.9	4.6
	407/371	0.5125	0.5562	**0.0221**	1.5	0.8	4.1
CA	407/407	0.8129	0.4707	**0.0051**	0.9	1.2	1.8
	407/343	0.5155	0.9920	**0.0073**	0.7	1.0	2.3
DCA	391/391	**0.0125**	**0.0043**	**0.0003**	0.5	1.6	5.6
	391/343	**0.01858**	**0.00745**	**0.0005**	0.5	1.8	6.0

* in bold, *p*-value < 0.05, underlined *p*-value < 0.01; [a] S/N too low for good integration.
